# Idiosyncratic changes in spring arrival dates of Pacific Northwest migratory birds

**DOI:** 10.7717/peerj.7999

**Published:** 2019-11-07

**Authors:** W. Douglas Robinson, Christina Partipilo, Tyler A. Hallman, Karan Fairchild, James P. Fairchild

**Affiliations:** 1 Department of Fisheries and Wildlife, Oregon State University, Corvallis, OR, USA; 2 Philomath, OR, USA

**Keywords:** Migration timing, Songbirds, Citizen science

## Abstract

Shifts in the timing of bird migration have been associated with climatic change and species traits. However, climatic change does not affect all species or geographic locations equally. Climate in the Pacific Northwest has shifted during the last century with mean temperatures increasing by 1 °C but little change in total annual precipitation. Few long-term data on migration phenology of birds are available in the Pacific Northwest. We analyzed trends in spring arrival dates from a site in the Oregon Coast Range where nearly daily inventories of birds were conducted in 24 of 29 years. Several species showed statistically significant shifts in timing of first spring arrivals. Six of 18 species occur significantly earlier now than during the initial phase of the study. One species arrives significantly later. Eleven show no significant shifts in timing. We associated trends in spring migration phenology with regional climatic variables, weather (precipitation and temperature), traits of species such as migration strategy, foraging behavior, diet, and habitat use, and regional trends in abundance as indexed by Breeding Bird Survey data. We found no set of variables consistently correlated with avian phenological changes. Post hoc analyses of additional climate variables revealed an association of migratory arrival dates across the 18 species with rainfall totals in northern California, presumably indicating that songbird arrival dates in Oregon are slowed by spring storm systems in California. When only the six species with the most strongly advancing arrival dates were analyzed, winter maximum temperatures in the preceding three winters appeared consistently in top models, suggesting a possible role for food availability early in spring to promote the survival and successful reproduction of the earliest-arriving birds. However, additional data on food availability and avian survival and reproductive success are required to test that hypothesis. Despite the appearance of some climate variables in top models, there remains a mismatch between strongly advancing arrival dates in some songbirds and a lack of clear directional change in those climate variables. We conclude that either some previously unrecognized variable or combination of variables has affected the timing of migration in some species but not others, or the appearance of statistically significant directional changes over time can occur without being driven by consistent environmental or species-specific factors.

## Introduction

Migratory birds track favorable environmental conditions across their annual life cycle. Neotropical migrants, for example, breed in north temperate areas of North America during spring and summer before traveling to lower latitudes and avoiding the temperate winter. Even short-distance migratory birds, which breed north then move into the southern United States and northern Mexico during winter, respond to changing seasons. Although endogenous rhythms and cues from changing day length influence the timing of spring migration, birds also adjust the timing and speed of their migration to weather and other environmental conditions along the migratory route (Ahola et al., 2004; Marra et al., 2005; Gordo, 2007).

Cues along migratory routes that are correlated with conditions on breeding grounds include the timing and degree of spring leaf-out (Kelly et al., 2016). Recently, timing of spring leaf production has advanced and has been associated with earlier, warmer springs (Mayor et al., 2017). Since many migratory bird species are insectivorous and consume leaf-eating arthropods, birds arriving soon after leaf-out when insect numbers are increasing may experience demographic benefits (Miller-Rushing et al., 2010). Evidence from many geographic regions indicates that migratory birds have adjusted the timing of their spring migration in recent decades (Horev, Yosef & Pinshow, 2010; DeLeon, DeLeon & Rising, 2011). Yet, data can be influenced by sampling error and patterns of phenological change are inconsistent across geographic regions and species (Rubolini et al., 2007; Knudsen et al., 2011; Seavy et al., 2018).

To evaluate possible shifts in timing of migration, information on spring arrival dates of migratory birds gathered over fairly long periods of time is necessary. In addition, evidence that surveys were conducted daily during migration periods is required, otherwise sampling bias can drive the appearance of shifts in migratory schedules (Ptaszyk et al., 2003; Van Strien et al., 2008). For example, many bird migration data are gathered by amateur bird watchers who have a greater tendency to report observations from weekends than weekdays (Miller-Rushing, Primack & Stymeist, 2008a; Vitale & Schlesinger, 2011). If migration schedules shift by just a few days over time, daily surveys are necessary to detect such differences. Unfortunately, such data are rare, especially in the Pacific Northwestern United States (Gordo, 2007; Seavy et al., 2018), a region with large numbers of migratory birds but historically few bird observers.

Temperature has increased in the Pacific Northwest by about 1 °C while rainfall amounts have been relatively stable (Mote, 2003; Mote et al., 2014). Hydrological and plant phenological variables associated with arrival of spring indicate an advance of 1–2 weeks over the last few decades in western North America (Cayan et al., 2001). A number of ecological changes have been observed in the Pacific Northwest in response to these climatic changes, including earlier migration of sockeye salmon (*Oncorhynchus nerka*) in the Columbia River (Crozier, Scheuerell & Zabel, 2011), and earlier blooms in lilac and honeysuckle (Cayan et al., 2001). Whether or not migratory birds have responded similarly remains unclear.

Associations with changing climatic variables or ecological traits of species are not required to explain shifts in dates of first detection each spring. Dates of first detection could be influenced by changes in regional population size (Tryjanowski & Sparks, 2001; Sparks, Roberts & Crick, 2001; Miller-Rushing et al., 2008b). The first arrivals each spring are difficult to detect because those arriving birds are, by definition, very rare, so the probability of encountering first arrivals is low. If the regional population size of a species is increasing over time, the probability of detecting arrivals on a particular date each year should increase. The probability of detecting them earlier should increase as well, providing the appearance of a shift in arrival dates. Likewise, if regional population sizes are decreasing, an apparent shift toward later arrival dates could be attributed to reduced chances of discovery across the arrival time period. Some researchers have also suggested that analyses of first arrival dates are the most susceptible to sampling error and recommend use of third arrival dates but a careful comparative analysis indicated that both approaches produce similar results (Ptaszyk et al., 2003).

We analyzed a unique 29-year-long survey of birds from the Oregon Coast Range. Presence–absence data were collected nearly daily for all bird species, providing information on temporal patterns of spring arrival dates. Our objectives were, first, to examine the data for trends in arrival dates and, second, if significant trends were detected, to associate trends and migratory arrival dates with environmental variables and ecological traits of the bird species so as to evaluate possible explanations for migration phenology. Besides local temperature and rainfall measurements, we included indices of regional climatic conditions because prior studies revealed correlations of large-scale climate patterns, such as the North Atlantic Oscillation (Hüppop & Hüppop, 2003; Vähätalo et al., 2004) on spring migration phenology. We included data from February, March and April for the El Niño Southern Oscillation (ENSO) and the Pacific Decadal Oscillation (PDO; Newman et al., 2016), two related large-scale phenomena that influence weather patterns along coastal western North America during the time that our species are migrating northward. We also included a multivariate index of several ENSO-related environmental variables, the multivariate ENSO index (MEI; Wolter & Timlin, 2011). Because changes in winter temperatures might influence spring arrival dates (Swanson & Palmer, 2009), we also incorporated measures of local weather including maximum winter temperature (December through February). We evaluated associations with temporal change in regional population size by comparing the trends in arrival dates with Breeding Bird Survey (BBS) data as an index of change in regional population sizes. Our overall goals were to quantify phenological changes in the migratory arrival dates and to evaluate explanations of patterns in arrival dates by associating those changes with climatic data, regional population sizes, and species’ traits.

## Methods

In 1985, Jim and Karan Fairchild began keeping daily lists of bird species detected on their 32-ha farm, Alder Spring, near Philomath, Benton County, Oregon (44.49265°N, 123.46249°W). Alder Spring is situated in the eastern edge of the Western hemlock vegetation zone of the Oregon Coast Range, transitioning into the Oregon White Oak (*Quercus garryana*) plant communities of the interior Willamette Valley (Franklin & Dyrness, 1998). Alder Spring is currently 90% forested. Ten percent remains in grassland, maintained around a single residence. The forest is composed mainly of mature Douglas-fir (*Pseudotsuga menziesii*), with scattered occurrences and overstory inclusions of golden chinquapin (*Chrysolepis chrysophylla*), western hemlock (*Tsuga heterophylla*), Oregon white oak, and grand fir (*Abies grandis*). Pacific dogwood (*Cornus nutallii*), cascara (*Rhamnus purshiana*), and Pacific madrone (*Arbutus menziesii*) fill in the intermediate canopy. Hazel (*Corylus* sp.), ocean spray (*Holodiscus discolor*), and vine maple (*Acer circinatum*) dominate the tall shrub layers, while salal (*Gaultheria shallon*), sword (*Polystichum* sp.) and bracken fern (*Pteridium* sp.) dominate the low shrub layer of the forest. There is a high degree of heterogeneity in the forest, both vertically and horizontally. Areas of larger, widely-spaced trees give way to dense patches of pole-size and smaller younger trees, and significant canopy openings can be found throughout the forest. Even-aged Douglas-fir forest management now dominates the surrounding landscape. Clear-cutting adjacent to Alder Spring occurred in 1978, 1988, 2000 and 2007, each time affecting about 13% (0.35 km) of the property ownership boundary (3.9 km).

Two light forest thinning events (<9% of the standing board foot volume) in 1989 and 2006 within Alder Spring selectively removed Douglas-fir while releasing other tree species and tall shrubs. The non-forested area near the residence is maintained through mowing, pruning and removal of encroaching trees. Since 2000 some low shrubs suitable for dry sunny sites (*Manzanita*, *Ceanothus*, *Mahonia*) have been introduced. Overall, the site is very similar to the surrounding landscape for hundreds of square kilometers.

All bird species detected each day were noted. The number of observers varied from one to three. Birds were not observed at a consistent time of day, but were usually noted as they were detected during outdoor activities. An evening family ritual of tallying the species detected each day formed the basis for noting sightings in field books. Although no record of effort per day was kept, across the study period data were collected on 97% of the possible days during the spring migratory period (21 March to 20 June) and no consistent pattern of “missing” days across years was noted. All data are archived in eBird (eBird.org) under the site name Alder Spring.

We analyzed first arrival dates of migratory species detected on the property from 1985 to 2014. A notebook containing data from 1989 to 1993 was lost, removing those data from our analyses. To be included, a species had to have been detected in at least 12 of the 25 years. Most species included in our analyses were detected annually and all bred on or near the property. We assigned species to guilds based on primary breeding habitat and diet. Breeding habitats were coniferous woodland and deciduous woodland or shrubby second-growth, the latter of which was primarily composed of deciduous species. Dietary categories included insectivore, frugivore/granivore, and nectarivore. Although we used these three categories of dietary guild, nearly all species are actually omnivorous during the breeding season. We also grouped species based on typical foraging height above ground (terrestrial and understory, midstory and canopy or above). We categorized species as short-distance migrants and long-distance migrants. Short-distance migrants are those species that do not typically over-winter in the Coast Range of western Oregon but move south into California or other areas of the western United States. Long-distance migrants typically over-winter in Mexico and Central America with some species traveling as far as South America. Identification of species trait information was based on personal observations and the Birds of North America (birdsna.org).

We examined associations of the following local weather variables (from the Corvallis Weather Bureau station located eight km from Alder Spring; ncei.noaa.gov) with first spring arrival dates: April and total annual rainfall; April average temperature; April minimum low temperatures; maximum and minimum temperatures in the preceding winter (defined as December, January and February) and the 3-year running average of winter temperatures prior to each spring. Climate variables included indices of the PDO and of the ENSO and MEI for February, March and April, the time during which our species would be departing their winter sites and migrating northward. The PDO captures regional climatic characteristics influenced by sea surface temperatures north of 20°N in the North Pacific Ocean (Mantua et al., 1997; Mantua & Hare, 2002; Newman et al., 2016; http://research.jisao.washington.edu/pdo/PDO.latest.txt). In general, negative values are associated with cooler and wetter months and positive values occur during warmer and drier months along the northern West Coast of the United States. ENSO influences weather systems along the western coast of North America and therefore may influence migration timing of birds. Generally speaking, cooler sea-surface temperatures (La Niña) lead to wetter conditions and more spring storms in the Pacific Northwest whereas warmer sea-surface temperatures (El Niño) produce drier conditions there and divert spring storms southward into northern California (Fierro, 2014). MEI is a composite index of five variables associated with ENSO seasonality (Wolter & Timlin, 2011). After analyses revealed some influence of PDO indices suggesting possible relationships with migratory arrival dates and conditions during passage through California, we added monthly (February, March and April) average rainfall data from Sacramento, California, to our models. Rainfall in northern California is positively correlated with warm sea-surface temperatures, values which are reflected in PDO measurements (Fierro, 2014). We hypothesized that a flow of storm systems through northern California might delay spring arrival of migrant birds in Oregon, whereas clear and dry weather in California would accelerate passage, creating earlier arrival dates. The exact wintering areas of birds breeding at Alder Spring is unknown, so identifying climate signals in species-specific wintering areas was not attempted.

We compared trends in first spring arrival dates of each species with BBSs at two spatial extents: a national scale using all BBS data available and a regional scale using data from BBS routes in the Oregon Coast Range. None of the Oregon Coast Range BBS routes had continuous data during the time period of our study. Five routes were surveyed from 8 to 23 of the 25 years. Those included were Nicolai Mountain, Timber, Trask Summit, Riley Peak, and Salado, all within 150 km of Alder Spring.

To quantify statistical trends in first arrival dates over time, we first used linear regression conducted in JMP version 12.2 (SAS Institute Inc., Cary, NC, USA). When necessary, we first transformed data to meet assumptions of normality. We calculated temporal trends in first arrival dates for each species as the slope of the regression between year and first arrival dates. This first step was designed to understand if any species were arriving significantly earlier or later. We then compared those trends with the categorical species trait data (diet, foraging behavior, habitat type, height above ground while foraging, and short- versus long-distance migrant as defined by their primary wintering geographic zone) using ANOVA. Once we determined that some species were indeed arriving at different times than they arrived in the late 1980s, but we were unable to discover any strong associations with categorical species traits, we used model selection to examine associations of climatic variables with arrival dates in R (Version 3.5.3; R Development Core Team, 2013). To do so, an enlarged set of climatic variables were included in models evaluated by BIC with the dependent variable being species’ arrival dates. The enlarged set of climatic variables included local rain and temperature data during the migratory months as well as during winter months (see Discussion and Supplemental Files). By first running separate model sets for each species, we allowed species to respond differently to climatic variables within our models without the need for complex interactions. We then evaluated all species together in the same model, with species included as a variable, to determine if generalizable associations with climatic variables were present in the data. Finally, we grouped the six species exhibiting the strongest shifts toward earlier arrival dates and evaluated overarching effects of climatic variables on arrival dates of those species. We used BIC instead of AIC because our objective was to explain patterns within the data rather than predict values outside the range of our data (Schmueli, 2010).

## Results

**Figure 1:**
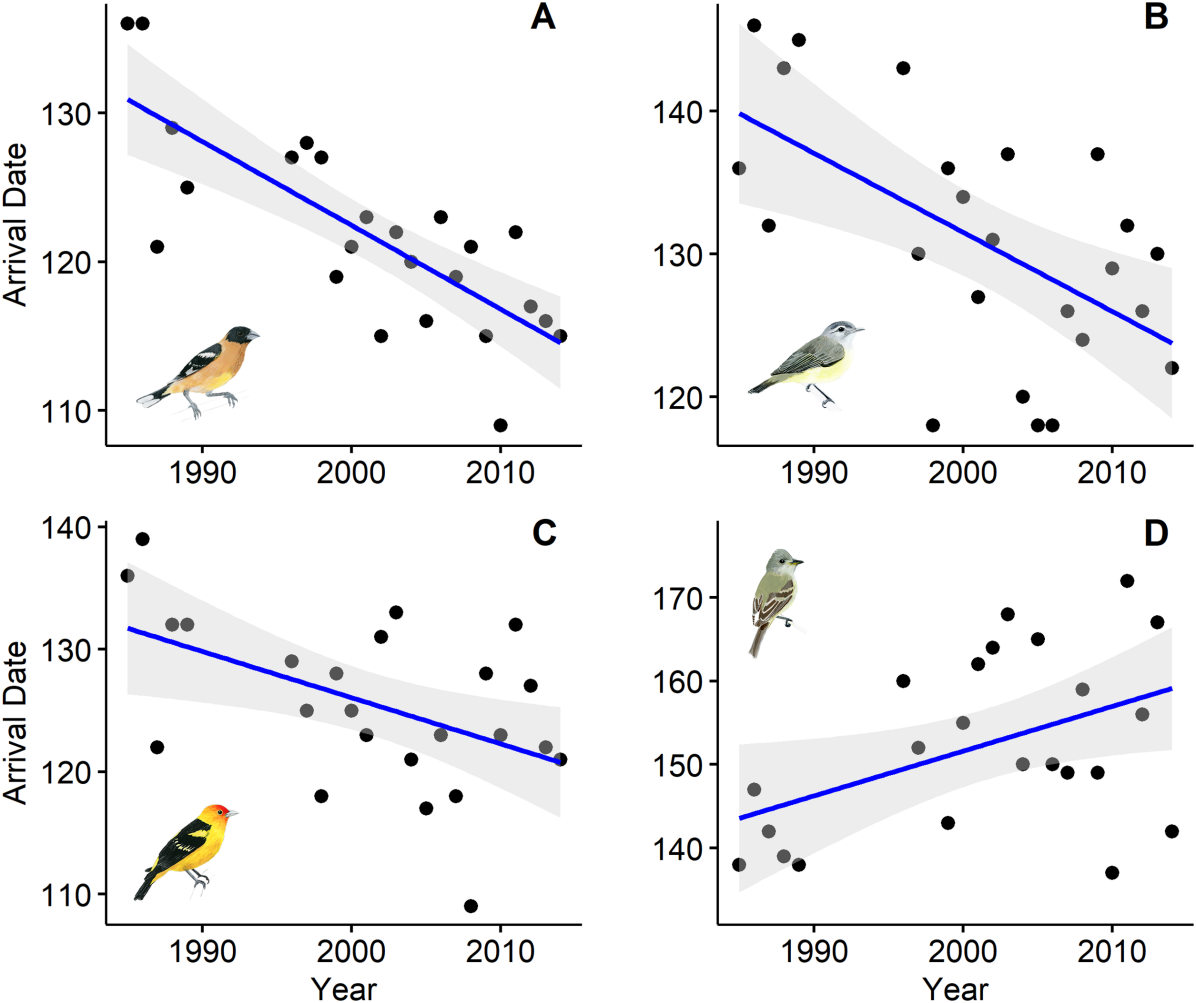
First spring arrival dates of four bird species at Alder Spring, Benton County, Oregon. The linear temporal trend is shown by a blue line (confidence intervals in gray). Arrival dates are day of year (May 1 = 121). Black-headed Grosbeak (A), Warbling Vireo (B), and Western Tanager (C) now arrive 12–16 days earlier than 30 years ago and Willow Flycatcher (D) now arrives 2 weeks later. Artwork credit: Tara Rodden Robinson.

Ten of the 18 species showed a trend toward arriving earlier each spring, but only six were statistically significantly earlier arrivals (Table 1). Spring arrival dates of some species advanced during the 25-year study by as much as 2.2 weeks (Fig. 1). Black-headed Grosbeaks, for example, arrived 11 May, on average, in the late 1980s, but now arrive 25 April (Table 2). Willow Flycatcher showed the strongest shift toward later arrivals with average arrival in the late 1980s on 24 May and a predicted arrival date of 8 June at the end of our study.

**Table 1:** Relationships of first spring arrival date with year for 18 bird species detected at Alder Spring, Philomath, Oregon, 1985–2014. Species are sequenced alphabetically.

Species	Slope	*R*^2^	*p*
American Goldfinch *Spinus tristis*	–0.438	0.339	0.0028
Black-headed Grosbeak *Pheucticus melanocephalus*	–0.564	0.608	<0.0001
Black-throated Gray Warbler *Setophaga nigrescens*	0.250	0.155	0.0569
Cedar Waxwing *Bombycilla cedrorum*	0.001	0.001	0.9928
Hammond’s Flycatcher *Empidonax hammondii*	0.204	0.054	0.2854
Hermit Warbler *Setophaga occidentalis*	0.022	0.002	0.8105
House Wren *Troglodytes aedon*	–0.963	0.495	<0.0008
MacGillivray’s Warbler *Geothlypis tolmiei*	0.185	0.031	0.4116
Olive-sided Flycatcher *Contopus borealis*	–0.600	0.263	0.0122
Orange-crowned Warbler *Oreothlypis celata*	0.005	0.002	0.9798
Pacific-slope Flycatcher *Empidonax difficilis*	–0.182	0.045	0.3815
Rufous Hummingbird *Selasphorus rufus*	–0.019	0.002	0.8571
Swainson’s Thrush *Catharus ustulatus*	–0.038	0.007	0.6964
Violet-green Swallow *Tachycineta thalassina*	–0.200	0.013	0.5985
Warbling Vireo *Vireo gilvus*	–0.555	0.343	0.0026
Western Tanager *Piranga ludoviciana*	–0.378	0.247	0.0134
Willow Flycatcher *Empidonax traillii*	0.537	0.209	0.0281
Wilson’s Warbler *Cardellina pusilla*	0.192	0.153	0.0709

**Table 2:** Predicted arrival dates in 1989 and 2014 for 18 bird species. Predictions are based on linear regressions of the first arrival dates across the span of the study at Alder Spring, Philomath, Oregon. Species are ordered based on predicted arrival date in 1989. See Table 1 for scientific names.

Species	Predicted 1989	Predicted 2014
Rufous Hummingbird	17 March	6 March
Violet-green Swallow	28 March	22 March
Orange-crowned Warbler	5 April	5 April
Black-throated Gray Warbler	9 April	17 April
Wilson’s Warbler	19 April	24 April
MacGillivray’s Warbler	20 April	25 April
Hermit Warbler	26 April	25 April
American Goldfinch	28 April	15 April
Pacific-slope Flycatcher*	30 April	25 April
Hammond’s Flycatcher	3 May	9 May
Black-headed Grosbeak	11 May	25 April
Western Tanager	12 May	1 May
Swainson’s Thrush	13 May	12 May
House Wren	15 May	17 April
Warbling Vireo	20 May	4 May
Cedar Waxwing	22 May	22 May
Willow Flycatcher	24 May	8 June
Olive-sided Flycatcher	29 May	11 May

**Note:**

* Pacific-slope Flycatcher dates are based on detections beginning in 1996.

We found no strong associations of trends in arrival dates with our measurements of habitat use, diet, over-wintering sites, or regional population trends from the BBS. Trends in arrival dates were not associated with wintering geographic location (*F* = 0.67, *df* = 2,17, *p* = 0.52), type of foraging (sally versus glean) (*F* = 0.46, *df* = 1,17, *p* > 0.5), choice of habitat interior versus edge (*F* = 0, *df* = 1,17, *p* > 0.99), foraging height (*F* = 0.16, *df* = 2,17, *p* = 0.85) or preference for coniferous versus deciduous habitat (*F* = 1.58, *df* = 1,17, *p* = 0.22). Temporal spring arrival trends among the 18 species were not statistically significantly related to national or regional BBS population trends (all comparisons, *p* > 0.3).

We then evaluated climate variables to determine if any directional trends were apparent. In the Corvallis area, the only statistically significant change was average April temperature, which was 1 °C cooler by the end of the study (*F* = 4.58, *df* = 1,21, *p* = 0.044). We found no significant trends in temperatures in other spring months or rainfall in the Corvallis data during the span of our study. We found no clear trends in winter temperatures or their 2-year or 3-year running averages. At the local level, arrival timing was not significantly associated with rainfall amounts during April or the entire year in any of the 18 species. We found no significant correlations of species arrival date trends with average temperatures during April nor with the highest maximum or lowest minimum temperatures during April. Regionally, no significant trends in ENSO or MEI indices were found. PDO, however, shifted significantly during the study from positive values toward negative values at an average rate of –0.05 units per year and trended significantly in all 3 months we studied: February (*F* = 5.91, *df* = 1,22, *p* = 0.024), March (*F* = 8.31, *df* = 1,22, *p* = 0.009) and April (*F* = 4.80, *df* = 1,22, *p* = 0.039).

Few significant statistical relationships between species traits, local weather variables, and arrival dates were detected, yet six species were clearly arriving earlier and one much later. We therefore investigated to what degree arrival dates of all study species were associated with climatic variables. When all species were included, Sacramento rainfall during March occurred in four of the top models, which held 94.3% of model weight. MEI occurred in three of the top five models. Sacramento rainfall and MEI are only weakly negatively correlated (–0.12). In general, years with greater rainfall in northern California were associated with later arrival dates at Alder Spring. Individual species models included a highly inconsistent array of variables, with no obvious patterns in the importance of particular climatic variables (Supplemental Files).

Finally, we asked, for the six species arriving earlier, were there associations with any of the climate variables? Here, we found that the 3-year moving average of maximum winter temperature in the study area was in the six top models; those top models accounted for 99.1% of the model weight (Table 3). No other climatic variables were consistently included in the top models.

**Table 3:** Model selection results for analyses of spring arrival dates by the six species showing the strongest shift in arrival date since 1985. Top models accounting for 99.1% of model weight are shown. Full table included in Supplemental Materials.

Variables included	*df*	Log likelihood	BIC	Delta	Weight
WinterMaxT_3MovAvg__species	8	–480.111	999.641	0	0.490
WinterMaxT_3MovAvg__ MEI__species	9	–478.674	999.641	2.052	0.175
WinterMaxT_3MovAvg__ SACrainMar__species	9	–478.881	1002.108	2.466	0.142
WinterMaxT_3MovAvg__ CorAvgT__species	9	–479.394	1003.134	3.492	0.085
WinterMaxT_3MovAvg__ PDO__species	9	–479.846	1004.038	4.396	0.054
WinterMaxT_3MovAvg__ WinterMinT_3MovAvg__species	9	–480.081	1004.507	4.8661	0.043

## Discussion

Changes in spring arrival dates of migratory birds from 1985 to 2014 in the Oregon Coast Range were idiosyncratic with respect to species and the variables we analyzed. Some species arrived more than 2 weeks earlier in recent years or arrived up to 1 week later, whereas most species showed no change in first arrival date. We found no strong associations of arrival dates with species’ dietary preferences, preferred breeding habitat, general location of wintering areas, or national, regional or local trends in abundance as indexed by BBS results. First arrival dates were not strongly associated with local weather variables such as annual rainfall nor rain totals during April, the month when most species were arriving.

Having found little explanation for clearly strongly shifting phenology in seven of the 18 species, we then conducted additional analyses to assess influence of climatic variables on arrival dates. When all 18 species were analyzed, rainfall amounts in northern California (as indexed by Sacramento monthly rainfall during spring) appeared in top models as potential factors influencing arrival dates in Oregon. Generally speaking, in springs with more rain south of Oregon, migrants were delayed arriving in Oregon whereas in years with less rain in northern California migrants arrived earlier. We hypothesize that inclement weather impeded movements of migrants, however we cannot exclude the alternate explanation that migrants delayed timing of northward passage to take advantage of food resources available in California owing to vegetational responses to spring rains. Despite the appearance of Sacramento rainfall in top models, we found no directional trend over time in Sacramento spring rainfall totals, which renders interpretation of directional shifts in migratory arrival dates in Oregon problematic.

We then restricted our analyses to only the six species arriving significantly earlier over time. In those models, the 3-year running average of winter maximum temperatures preceding the arrival date consistently appeared in top models. It has previously been suggested that winter temperatures might influence spring arrival dates (Swanson & Palmer, 2009). In particular, wetland species that respond to early thawing of frozen habitat are thought to be especially plastic in their movement schedules, in addition to short-distance migrants that may experience stronger correlations between weather at wintering and breeding sites than long-distance migrants wintering at very distant sites experience (Gordo, 2007). In our study, all species are terrestrial songbirds or near-songbirds, so thawing plays no role, and most are considered long-distance migrants, wintering in Latin America. The appearance of winter maximum temperature in our models may suggest that warmer winters allow the earliest-arriving individuals to survive and possibly to gain a reproductive advantage that is promulgated forward over time. However, we know of no data on influence of winter temperatures on availability of songbird food items in the Pacific Northwest, nor are we aware of any data showing differential survivorship of early- versus late-arriving songbirds in our region. Despite the intellectual appeal of this hypothesis of differential survivorship, similar to the lack of temporal directional trends in Sacramento rainfall totals, we found no obvious directional trends over time in winter maximum temperatures in our study region. Thus, we conclude that a parsimonious explanation for timing of spring arrival dates includes an influence of winter temperatures, but that influence does not seem to explain the significant advancement of arrival dates in six species.

We know of no other long-term studies of spring migration phenology in the Pacific Northwest, so we cannot yet determine if the idiosyncratic patterns are unique to the region. However, the result that magnitude of phenological changes over time are often species-specific is an increasingly common result (Végvári et al., 2010). A banding station study of migration timing of five songbird species in northern California also found idiosyncratic patterns weakly associated with regional climate indices (Barton & Sandercock, 2018). Some studies in other regions have found associations of migratory distance with spring arrival dates, generally revealing that short-distance migrants appear to respond more quickly to warming environmental conditions and arrive earlier than long-distance migrants that rely mostly on changes in day length to initiate migration (Gwinner, 1996; Gordo, 2007). Yet, even long-distance migrants may be able to adjust arrival dates on breeding grounds by responding to conditions along their migratory route (Marra et al., 2005). Compared with studies from elsewhere in western North America, ours seems to be the only study revealing such an eclectic mix of temporal patterns of arrival dates among species.

We are unaware of other obvious ecological or life history variables that could be associated with patterns of spring arrival dates among the diverse set of species included in our study. The possibility that the trends are spurious, generated by systematic observer errors, seems highly unlikely. The same observers collected the data at the same site for the duration of the study without intent to analyze the data. If improvements in identification skills over time were influential, one would expect earlier arrival dates of the species generally considered most difficult to identify, such as the *Empidonax* flycatchers. Yet, two of those three species arrived much later toward the end of the study. Changes in observer skill or systematic observer bias appear to be unlikely explanations.

We cannot completely exclude the possibility that the species arriving earlier have larger regional population sizes than they had 30 years ago. We used BBS trends as an index of changing population sizes. If the trends are not reflective of actual regional population sizes, then our test of the population size influence may be ineffective. We used both national and regional BBS data, finding no clear associations with spring arrival dates. However, the influence of continental-scale trends may be too diffuse to find relationships with small-scale regional population sizes and the regional BBS data could be too incomplete. The five BBS routes in the Oregon Coast Range were surveyed from 7 to 23 times during the span of our study. Overall, without another comprehensive set of surveys with which to compare BBS data (which does not exist for this relatively poorly studied region of North America), we cannot evaluate the adequacy of BBS trend data for indexing regional population change over time.

Most published studies evaluating shifts in migration timing have found significant changes in at least some of the species studied. Few have been unable to find statistically significant predictors of change. At least one study has reported complex patterns like ours and showed inconsistent shifts among species as well as no clear associations with species characteristics (Van Buskirk, Mulvihill & Leberman, 2009). Most published studies among North American birds have found at least a few species with statistically significant advances, yet most of the species (40–88%) show no significant advances (Barton & Sandercock, 2018). Van Buskirk, Mulvihill & Leberman (2009) argue that an emphasis on analysis of first spring arrival dates reveals greater advance in arrivals than analyses including the entire distribution of species detections during a migratory period. Although our data lack counts of individuals for each species during spring, preventing analysis of entire distributions of abundance at our site, it is clear that patterns of arrival are not consistently advancing across species, but are instead quite idiosyncratic.

## Conclusions

Seven of 18 migratory bird species showed substantial changes in migration schedules at our Pacific Northwest study site. Despite the changes, none of the typical suite of species traits or weather variables were consistently identified as influential on spring arrival dates. From post hoc analyses, we found associations between the occurrence of spring weather systems in northern California, which presumably slowed rates of passage northward during inclement weather, and the average maximum winter temperatures in preceding winters. Warmer winters may allow the earliest-arriving birds to encounter sufficient food supplies and begin breeding earlier but, like most study areas, few data on food availability or demographic variables are available for the Pacific Northwest. Counterintuitively, despite advancing spring arrival dates in seven bird species, there was no clear directionality in northern California rainfall amounts nor in average winter temperatures in our study region. We conclude that identifying mechanisms responsible for shifting migration schedules will require additional investigation of songbird food phenology, comparisons of demographic consequences of early- versus later-arriving individuals, better measurements of population sizes through time and greater understanding of linkages between climate dynamics and spring green-up of vegetation.

## Supplemental Information

10.7717/peerj.7999/supp-1 Supplemental Information 1 Model selection results for all species.

10.7717/peerj.7999/supp-2 Supplemental Information 2 Model selection results for the six species arriving significantly earlier.

10.7717/peerj.7999/supp-3 Supplemental Information 3 Data matrix used in analyses of arrival dates versus environmental variables.

10.7717/peerj.7999/supp-4 Supplemental Information 4 Data matrix for arrival date trends, species traits and Breeding Bird Survey trends.

10.7717/peerj.7999/supp-5 Supplemental Information 5 Individual species model results for American Goldfinch.

10.7717/peerj.7999/supp-6 Supplemental Information 6 Individual species model results for Black-headed Grosbeak.

10.7717/peerj.7999/supp-7 Supplemental Information 7 Individual species model results for Black-throated Gray Warbler.

10.7717/peerj.7999/supp-8 Supplemental Information 8 Individual species model results for Cedar Waxwing.

10.7717/peerj.7999/supp-9 Supplemental Information 9 Individual species model results for Hammond’s Flycatcher.

10.7717/peerj.7999/supp-10 Supplemental Information 10 Individual species model results for Hermit Warbler.

10.7717/peerj.7999/supp-11 Supplemental Information 11 Individual species model results for Macgillivray’s Warbler.

10.7717/peerj.7999/supp-12 Supplemental Information 12 Individual species model results for Orange-crowned Warbler.

10.7717/peerj.7999/supp-13 Supplemental Information 13 Individual species model results for Olive-sided Flycatcher.

10.7717/peerj.7999/supp-14 Supplemental Information 14 Individual species model results for Pacific-slope Flycatcher.

10.7717/peerj.7999/supp-15 Supplemental Information 15 Individual species model results for Rufous Hummingbird.

10.7717/peerj.7999/supp-16 Supplemental Information 16 Individual species model results for Swainson’s Thrush.

10.7717/peerj.7999/supp-17 Supplemental Information 17 Individual species model results for Violet-green Swallow.

10.7717/peerj.7999/supp-18 Supplemental Information 18 Individual species model results for Warbling Vireo.

10.7717/peerj.7999/supp-19 Supplemental Information 19 Individual species model results for Western Tanager.

10.7717/peerj.7999/supp-20 Supplemental Information 20 Individual species model results for Willow Flycatcher.

10.7717/peerj.7999/supp-21 Supplemental Information 21 Species codebook. Four-letter codes and English names of species included in the article.

## Additional Information and Declarations

### Data Availability

The following information was supplied regarding data availability: The raw data is available as Supplemental Files.
